# Health needs assessment tool for identifying the health issues among community residents with unmet needs

**DOI:** 10.1186/s41182-025-00713-9

**Published:** 2025-02-25

**Authors:** Yurie Kobashi

**Affiliations:** Global Exchange Center, Fukushima Medical University School of Medicine, Fukushima City, Fukushima 960–1295 Japan

**Keywords:** Health needs assessment, Community-based projects, Citizen science, Community ownership, Resource-limited setting

## Abstract

**Background:**

This study aimed to discuss the method for designing a community project, especially in a resource-limited setting, using a health needs assessment tool, the Community Health Issues Interview Sheet, for the design of a project in the initial phase.

**Methods:**

The Community Health Issues Interview Sheet was developed; this tool applied more than four field and modified based on feedback from researchers and stakeholders.

**Results:**

When researchers from outside a target community design a community project, there are three factors to consider: resource size, target population, and focused health issues. The interview sheet was aimed at identifying priority health issues among the target population.

**Conclusions:**

All community projects should be well-designed, and priority health issues should be assessed using a health needs assessment tool, such as the interview sheet in the initial project stage. Further education for researchers about project design in communities with resource-limited settings should be provided, and research on the experience of using the health needs assessment tool should be accumulated.

## Background

Citizen science refers to the participation of residents in the scientific process in collaboration with researchers to address and resolve complex local issues in the real world using scientific methods [1]. Citizen science in the health field has been spreading alongside advances in health equity [2]. According to a review article by Rosas et al., to address health equity through citizen science, the following five aspects are required: expanding the focus on topics essential to health equity, increasing the diversity of people engaged as citizen scientists, increasing citizen scientists’ participation in advanced research stages, developing novel technologies for citizen scientists to collect data related to health equity, and increasing the rigor of methods for assessing the impact of health equity [2]. In particular, for community-based projects on health issues, collaboration between diverse stakeholders has been emphasized, including public–private–people partnerships (4P); the participation of “people” is particularly vital [3]. Furthermore, community ownership is vital for cost effectiveness in resource-limited settings [4–6]. Therefore, community ownership is a crucial public health issue in citizen science, particularly in resource-limited settings.

Various countermeasures have been proposed for achieving community ownership in resource-limited settings. According to Singh, community participation can be divided into five stages, from “co-option” to “co-learning” [7]. Co-learning, which has the highest community contribution, is the only stage that can be considered as community ownership [7]. The previous literature has shown that three stakeholder types need to play a role for community projects to be carried out smoothly: those outside the community, communicators, and community representatives [8]. Community representatives were those who can directly and frequently hear the voices of the target population, those who live in or frequently visit the target area, and those with experience in the health field, among others; the opinions of community representatives are crucial to achieve community ownership [8]. Chi et al. summarized the vital things to fund or disseminate of projects that match the priorities of the community was that evaluators of projects changed their metrics of success [9]. However, there is little literature on how to design a project in its initial stages to be cost-effective, high-quality, and achieve community ownership in resource-limited settings.

The purpose of this study was to discuss the method for designing a community project, especially in a resource-limited setting, using a health needs assessment tool, the Community Health Issues Interview Sheet, for the design of a project in the initial phase.

## Methods

First, the methods for designing a community project were discussed. These methods were refined across several fields through various types of community projects [10–13]. Second, a health needs assessment tool, the Community Health Issues Interview Sheet, was proposed. This tool was developed in the aforementioned fields and projects. We applied the tool in more than four fields and modified it based on feedback from researchers and stakeholders. Finally, the usage of the Community Health Issues Interview Sheet was clarified.

## Results

### The project’s three arrows of design

**Fig. 1:**
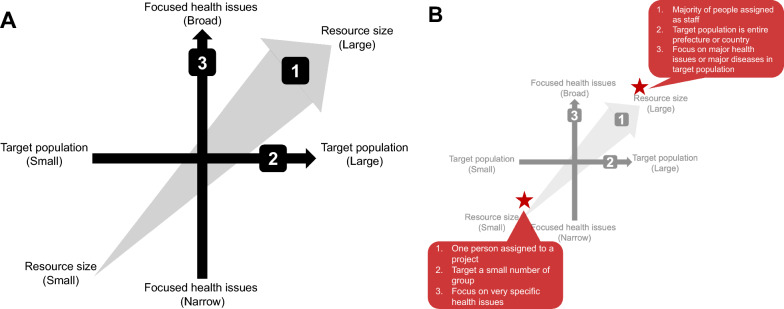
Project’s three arrows of design. **A** First arrow: resource size. Second arrow: target population. Third arrow: focused health issues. **B** Example of each project (the star shows each example of fictitious project)

When researchers from outside a target community design a community project, there are three factors to consider, which we convey using the concept of “arrows.” The first arrow concerns resource size, the second the target population, and the third focused health issues (Fig. 1).

At the start of project design, resource size might have already been decided, or it should be determined in the initial stage. The number of people or staff who engage in the project, the project duration, the capability of each staff member, and the specific resource setting and size should be determined first in the project design. For instance, if the project was conducted by two people over 2 years, the project could not have been designed to target a whole country’s population for all non-communicable diseases.

The target population can be determined based on the size of the resource (first arrow). The target population can be chosen based on one’s interest and the project aim; however, if the project is to be run by only two people, it may be focused on a small or narrow target group. If the project is to run by many people, it may be possible to focus on entire municipalities, prefectures, or countries.

The point of the third arrow, that is, focused health issues, may be defined by the first arrow (resource size) and the second arrow (target population). For instance, if a project has a small resource and a large target population, the third arrow should be narrowed and the focused health issues should be defined. However, if a project has substantial resources and a small target population, the target health issues may be broader.

### Health needs assessment tool: the Community Health Issues Interview Sheet

**Fig. 2:**
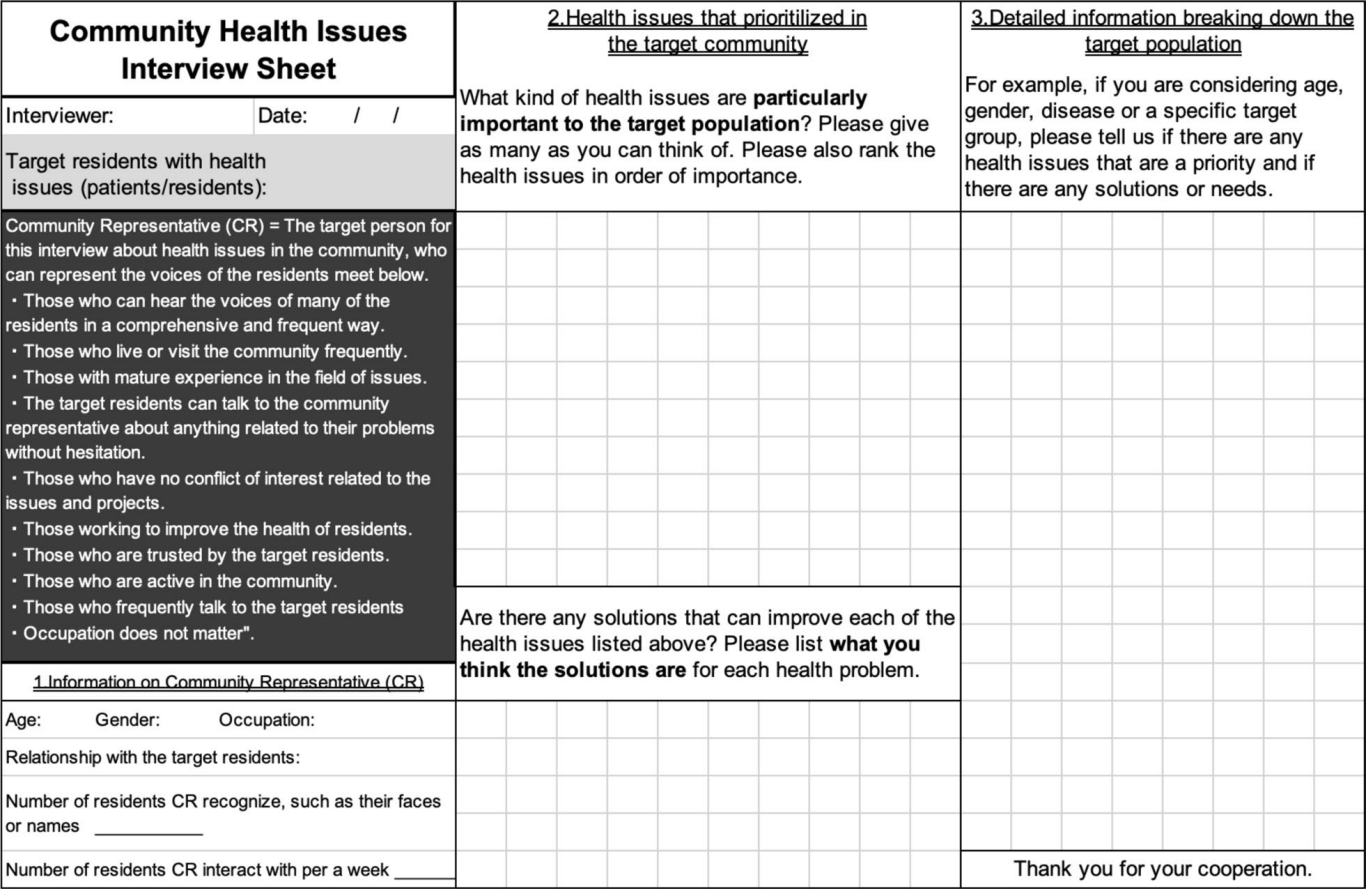
Health needs assessment tool (Community Health Issues Interview Sheet). How to use the Community Health Issues Interview Sheet: **1** first, discuss and decide which residents will be the target of this survey and fill in the gray area. **2** Next, carefully read the information inside the black area and decide the person (Community Representative) to interview. **3** Using the sheet created above, conduct an interview with the Community Representative to obtain responses to questions 1 to 3

The health needs assessment tool, the Community Health Issues Interview Sheet, may be useful for determining the optimal point of the third arrow. This interview sheet aims to identify priority health issues among a target population. Interviews are conducted with five to six community representatives who are familiar with the target population. From our experience and previous literature, we assumed that community representatives are: those who can directly and frequently hear the voices of many members of the target population, those who live in or frequently visit the target area, and those who with experience in the health field, among others [8] (Fig. 2).

We previously discussed the role of community representatives in our study in depth [8]; however, additional points of assessment are noted below. First, during the interviews, the interviewer could attempt to identify the representatives' motivations for their work. Community representatives often expressed their passion for improving community health, which was often reflected in their career paths. The interviewer occasionally observed voluntary actions, where representatives expanded their official duties to enhance the health of community members. Second, community representatives were highly cooperative during interviews about community health needs. Often, they could not conclude their interviews within the time limit due to the depth of their responses. They served as excellent educators for external researchers, passionately explaining how health issues affected their community and what actions were necessary to address these challenges. Notably, while individual community representatives provided valuable insights, relying on just one representative was insufficient. This is because the assessments from different community representatives sometimes varied significantly. However, when interviewing more than five community representatives, no additional opinions on prioritized health issues were likely to emerge.

The method for using the Community Health Issues Interview Sheet is outlined as follows (Fig. 2).

1. First, discuss and decide which population will be the target of the project based on the resource size and the project interests (identify the point along the second arrow).
2. Next, carefully read the information about the community representative in the interview sheet and list five to six community representatives who could be interviewed.
3. Using the interview sheet, interview the community representatives to obtain responses to questions 1 to 3 in the interview sheet.

Based on the resource size (first arrow) and target population (second arrow), the ideal size of the focused health issues is already defined, and priority health issues considering the ideal size should be identified from the interviews with community representatives using the Community Health Issues Interview Sheet (Fig. 2). This may help determine suitable target health issues for each project (third arrow).

## Discussion

In this study, we discussed a useful method for designing a project in a community and presented the Community Health Issues Interview Sheet as a health needs assessment tool.

This tool helps identify priority health issues among a target population, especially for outside researchers who are supervising research in the community for the first time. In our experience, when we have performed this kind of survey abroad, especially in rural areas of low–middle-income countries, we sometimes find that unexpected diseases have spread in the community, which extended knowledge based on clinical experience in the target area. For instance, gastroenteritis was found to be a severe health issue among rural Cambodia’s population. Previous studies have shown that the construction of health issues differed among the communities [14]. The viewpoints and voices of the community should be carefully reflected in each project using a health needs assessment tool.

To reduce poor design or lack of a viewpoint of target population community projects in communities with limited resources, all surveys should use this kind of health needs assessment tool. We have sometimes changed our research plan after a preliminary survey using the Community Health Issues Interview Sheet. Usually, we try to design the research according to the needs of the community; however, we may realize that the project does not meet their actual needs after a preliminary survey using the interview sheet. By increasing the projects matching to community needs, the education of those willing to be involved in a project in a resource-limited community can be improved. Increasing the number of people familiar with health needs assessment in the community may resolve this situation.

## Conclusions

For further cost-effective community projects, the project should be well-designed, and priority health issues should be assessed using a health needs assessment tool, such as the Community Health Issues Interview Sheet. Further education for researchers about project design in communities with resource-limited settings should be provided, and research on the experience of using the health needs assessment tool should be accumulated.

## Data Availability

No datasets were generated or analysed during the current study.
